# Menthol as a sustainable alternative anaesthetic for adult zebrafish (*Danio rerio*)

**DOI:** 10.1007/s11259-025-10860-3

**Published:** 2025-08-26

**Authors:** Luís Félix, Sandra M. Monteiro, Carlos Venâncio

**Affiliations:** 1 Centre for Research and Technology of Agro-Environmental and Biological Sciences (CITAB), University of Trás-os-Montes and Alto Douro (UTAD), Quinta de Prados, Vila Real, 5000-801 Portugal; 2 Institute for Innovation, Capacity Building and Sustainability of Agri-Food Production (Inov4Agro), UTAD, UTAD, Vila Real, Quinta de Prados 5000-801 Portugal; 3 Animal and Veterinary Research Centre (CECAV), UTAD, Vila Real, Quinta de Prados 5000-801 Portugal

**Keywords:** Anaesthesia, Aquaculture, Monoterpenes, Natural products, Sedation, Welfare

## Abstract

The use of anaesthetics has become pivotal in promoting ethical, sustainable, and responsible welfare practices in aquaculture and research procedures. However, species-specific reactions and side-effects have arisen from the use of the most common anaesthetics (MS-222 and eugenol). This study investigated the potential of menthol, a naturally derived and environmentally friendly compound, as an alternative immersion anaesthetic for adult zebrafish (*Danio rerio*) through concentration-response assays and the evaluation of cardiorespiratory, locomotion and aversive-related effects. Concentrations below 25 mg/L did not induce anaesthetic states while concentrations of 50 and 75 mg/mL were considered the most effective, as they successfully induced anaesthesia with induction and recovery times below 400 s. These values were comparable to those observed with MS-222. In addition, no mortality was observed at these concentrations despite the transient depression of the cardio-respiratory system. The video tracking analyses showed no significant behavioural changes for the lowest concentrations while relatively narrow behavioural safety margins were noticed for the highest concentrations (> 150 mg/L). In addition, concentrations above 75 mg/L induced aversive behaviours which do not support their use for fish anaesthesia. Based on these findings, a short-exposure (10-min) was conducted using menthol concentration of 50 mg/L, resulting in cortisol values equal to those of the control group. Overall, this study shows that a menthol concentration of 50 mg/L can be used as an alternative and more sustainable anaesthetic for zebrafish while higher concentrations may compromise fish welfare.

**Supplementary Information:**

The online version contains supplementary material available at 10.1007/s11259-025-10860-3.

## Introduction

Aquaculture has grown rapidly in recent years as a crucial means to meet the increasing demand for seafood while mitigating the impact on wild fish populations (Naylor et al. 2021). According to the United Nations Food and Agriculture Organization (FAO), around 89% of world fishery production is directly used as human food (FAO 2024). However, the intensification of aquaculture activities accompanied by the increase in basic and applied research with aquatic models (Supriya et al. 2022; Pouil et al. 2020; Mocho and von Krogh 2022), has raised concerns not only about the potential environmental consequences (Lin et al. 2022; Jiang et al. 2022) but also about the responsible and humane fish management (Brown and Dorey 2019). Mounting scientific evidence shows that fish species perceive and experience pain (Lambert et al. 2022) although the regulation of fish welfare is still underdeveloped in many regions (Toni et al. 2019; Browning 2023; Browman et al. 2019; Seibel et al. 2020). The lack of adequate welfare management strategies causes stress to the cultured fish, ultimately having major economic consequences (Toni et al. 2019). In this domain, anaesthetics emerged as a crucial ethical, sustainable, and responsible welfare promoter during aquaculture and research procedures (Schroeder et al. 2021; Sloman et al. 2019).

Tricaine methanesulfonate (MS-222) is an ester-type local anaesthetic agent commonly recommended anaesthetic in both aquaculture and research laboratories (Lidster et al. 2017; Topic Popovic et al. 2012; Carter et al. 2011). However, its species-specific effects and potential side effects have been reported (Readman et al. 2017; Carter et al. 2011). Aside from MS-222, some other anaesthetics have also been used or tested in fish (Martins et al. 2019), such as eugenol, a monoterpene alcohol that makes up over 80% of clove oil (Javahery et al. 2012; Fernandes et al. 2017). However, potential seizurogenic activity (Barbas et al. 2021) and serious lesions (Ayala-Soldado et al. 2023) have been reported. Over the last years, the anaesthetic activity of herbal anaesthetics (Hoseini et al. 2019) and other monoterpenes has been described in fish (Félix et al. 2023). For instance, research with menthol, a cyclic monoterpene alcohol of natural origin known for its ecological safety and low toxicity (Kamatou et al. 2013), has shown the effective anaesthesia of different fish species (Félix et al. 2023). More recently, menthol anaesthetic effects on zebrafish glucose homeostasis (Baesso et al. 2024), as well as its analgesic and anaesthetic properties in zebrafish larvae (Vieira et al. 2024; Rocha et al. 2024) have been described. The tropical teleost zebrafish (*Danio rerio*) has emerged as an excellent vertebrate model for a wide range of research areas, including developmental biology and toxicology (Choi et al. 2021; Cassar et al. 2020) and has also shown potential for applications relevant to aquaculture and fish welfare studies (Lee-Estevez et al. 2018; Ulloa et al. 2014; Jorgensen 2020; Piferrer and Ribas 2020). While not an aquaculture species per se, zebrafish provide valuable insights translatable to commercially important fish. However, the study of menthol as an alternative to MS-222 and eugenol using adult zebrafish has yet to be explored. Therefore, this work aimed to integrate physiological, behavioural, and cardiorespiratory responses in zebrafish anaesthetized with menthol and compare these responses to MS-222 and eugenol. The hypothesis was that zebrafish anaesthetized with menthol would exhibit similar responses across the anaesthesia stages (induction and recovery) when compared to the most commonly used anaesthetics. This would support the use of menthol as an effective alternative anaesthetic that contributes to the welfare of aquatic species.

## Methods

### Chemicals

Menthol (DL-menthol, 98%, 89-78-1) and eugenol (99%, CAS 97-53-0) were purchased from Alfa Aesar (Kandel, Germany), and MS-222 (ethyl 3-aminobenzoate methanesulfonate, 98%, CAS 886-86-2) was purchased from Merck (Algés, Portugal). Although different isomers of menthol exist, their composition was not considered for this study as the anaesthetic efficacy has been shown to be similar among them (Kasai et al. 2014). A stock solution of 89 g L⁻¹ was prepared for both menthol and eugenol using 90% ethanol, with final pH values of 7.2 ± 0.1 and 7.1 ± 0.1, respectively., while a stock solution of 1500 mg L^−1^ (MS-222) was prepared in distilled water and neutralized to pH 7.2–7.4 with sodium bicarbonate. All solutions were stored at 4 °C until further dilution.

### Animals and housing

**Fig. 1:**
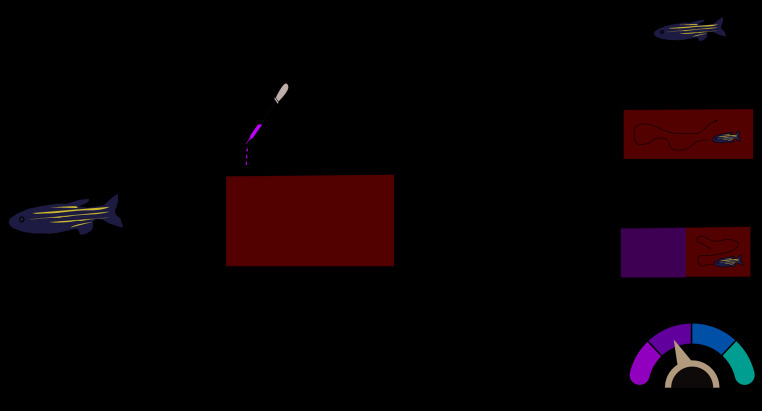
Schematic illustration of the experimental design and analysed endpoints. Adult zebrafish were individually and randomly distributed to MS-222, eugenol or varying concentrations of menthol to assess the anaesthetic profile, locomotion, and aversion behavioural. Based on these results, a 10-min exposure was conducted with 50 mg/L menthol concentration to assess potential cortisol alterations

Adult AB zebrafish (*Danio rerio*) were raised after hatching and maintained at a density of 2–3 fish/L (Andersson and Kettunen 2021) in the animal facilities of the University of Trás-os-Montes and Alto Douro (Vila Real, Portugal) as previously described (Felix et al. 2014). The 20-L glass tanks were supplemented through an open-system with aerated, dechlorinated, charcoal-filtered and UV-sterilized City of Vila Real tap water and maintained as follows: dissolved oxygen, 7.8 ± 0.7 mg/L; pH, 7.2 ± 0.3; temperature, 28.3 ± 0.5 °C; conductivity, 708 ± 128 µS/cm (86032 AZ meter); alkalinity, 28.0 ± 10.2 mg/L as CaCO_3_ (acid-base titration method); total hardness, 42.2 ± 13.6 mg/L as CaCO_3_ (EDTA titration); total ammonia nitrogen, 0.2 ± 0.2 mg/L; unionized ammonia, 0.0 ± 0.0 mg/L (Zamora-Garcia et al. 2021); nitrite, 0.1 ± 0.1 mg/L; and nitrate, 5.9 ± 5.1 mg/L (Carvalho et al. 1998). The fish tanks were maintained under a 14:10 light/dark cycle and animals were fed a nutritionally balanced diet (Zebrafeed, Sparos Lda., Portugal) twice a day (in the morning and in the afternoon). Fish (aged 12 to 18 months post-hatching) of both sexes (approximately 47% females, with a balanced sex ratio in each treatment group) with average body weight 0.36 ± 0.12 g and body length 3.38 ± 0.34 cm were included in the experiments, as no sex-specific anaesthetic effects have been reported on zebrafish for eugenol and MS-222 (Musk et al. 2020), although different sex recovery sensitivities have been described for essential oils (Seyidoglu and Yagcilar 2020). Animals were starved for 24 h before anaesthetic experiments (Neiffer and Stamper 2009). Animals were handled according to the standard protocols of the European Animal Welfare (Directive 2010/63/EU) and Portuguese (Decreto-Lei 113/2013 changed by Decreto-Lei n.º 1/2019) Legislation. In addition, the experimental protocol was ethically reviewed and approved by the ORBEA (Órgão Responsável pelo Bem-Estar Animal/Animal Welfare Body, 1805-e-CITAB-2023) and by the CE-UTAD (Ethics Committee, Doc49-CE-UTAD-2023) of the University of Trás-os-Montes and Alto Douro as well as by DGAV (Direção Geral de Alimentação e Veterinária/Directorate General for Food and Veterinary, 37744/25-S). A representation of the experimental procedure used in this work is found in Fig. 1.

### Anaesthetic profile determination

A total of 100 fish were used to evaluate induction and recovery times from menthol anaesthesia. The fish were removed from the stock tank and were transferred to acrylic containers (7.6 × 9.5 × 8.3 cm) containing 200 mL of the test solution. Different concentrations of menthol were tested (25, 50, 75, 100, 150 and 200 mg/L) as well as MS-222 (150 mg/L, (Collymore et al. 2014) and eugenol (80 mg/L, (Grush et al. 2004), as standard anaesthetic groups. Two control groups (anaesthetic/ethanol-free water and the maximum concentration of ethanol to dilute the highest concentration of menthol, 0.2%) were monitored under the same conditions. Anaesthetic baths were renewed for every animal, the treatment order was assigned using an online randomization tool (randomization.com), but no blind approach was applied due to the odour of the natural compounds. The fish were individually observed (*n* = 10 for each concentration), by the same observer until stage 3 anaesthesia was apparent (Sneddon 2012; Barbas et al. 2016). Briefly, the time to loss of equilibrium and erratic swimming despite maintaining the response to pressure on the caudal peduncle (stage A2) and the total loss of reflex activity and response to external stimuli (stage A3) were recorded with a digital stopwatch. To assess the response to pressure, a gentle pinch was applied to the caudal peduncle using tweezers, following standard anaesthesia evaluation protocols (Valentim et al. 2016). The maximum observation time for each stage was set at 10 min, after which the trial was terminated to prevent fish stress. After reaching stage A3, animals were removed from the anaesthetic bath and immediately placed in an equal plastic container with the same volume of clean water. The time to recover equilibrium (stage R1) and the complete regain of normal swimming pattern (stage R2) were also documented. The evaluation of recovery was not time-limited, but the health of the animal (ventilatory and cardiac frequency) was evaluated following a certain period, as described below. After complete recovery, animals were returned to their maintenance tanks and monitored for 96 h to check for potential mortality and behavioural change. These fish were reused for further experimentation if no discernible morphological and behavioural changes were detected over a period of one week, thereby reducing the overall animal use.

### Light cardiography and ventilatory signals

Immediately after reaching stage 3 anaesthesia, the fish were transferred to a glass petri-dish containing the same anaesthetic solution and a weighted sponge with a groove to hold the fish immersed with the ventral side up. A SZX7 stereomicroscope (Olympus, Tokyo, Japan) coupled to an EP50 digital camera (Olympus, Tokyo, Japan) was used to observe each fish. The room’s temperature was maintained at 27–28 °C. Following 30 s acclimation, a 1 min video was recorded using cold light illumination (Mousavi and Patil 2020). The heart rate was then determined by direct visual count (Pylatiuk et al. 2014) of the recorded video. The control animals were sedated for 5 min with 50 mg/L of MS-222 before analysis (Le et al. 2019b). The ventilatory frequency was also counted from the visual inspection of the recorded videos (Martins et al. 2018). If it was necessary to change the video speed, the open-source software VLC Media Player (version 3.0.20, VideoLAN, Paris, France) was used.

### Behavioural responses to anaesthesia

To evaluate the behavioural reactions to menthol anaesthesia and record the movement of fish during anaesthesia induction, a 3D video recording system was implemented as described elsewhere (Audira et al. 2018). Briefly, an acrylic box with a size of 10 × 10 × 10 cm was covered with non-reflective white sticker paper, leaving only the front face free to avoid distractions. A mirror was glued to the box’s lid and kept at an angle that allowed viewing the entire bottom of the box. Each fish (*n* = 10 per group) was individually transferred to this tank containing 400 mL of the anaesthetic concentrations described above. Immediately, 10-min high-definition videos were recorded by a smartphone camera (resolution: 1920 × 1080 pixel/30 frames per second) mounted in front of the tank. The 3D video files were then analysed using the TheRealFishTracker automated tracking system (Buske and Gerlai 2014) to capture XY and YZ coordinates for each fish. Behavioural variables such as total distance swam, average speed, meandering (movement without a fixed direction or path) and freezing time were extracted from the coordinates, as described by Audira et al. (2018), for the first 2 min, the mean time until the animal loses equilibrium. In addition, 3D swimming trajectory plots were obtained from the data using Origin software from OriginLab (Northampton, USA) as outlined elsewhere (Audira et al. 2018). The freezing (speed less than 1 cm/s), swimming (speed 1–10 cm/s), and rapid movement (speed higher than 10 cm/s) of zebrafish were assessed from the 3D plots as described by Audira et al. (2018). A total of 100 random adult fish were used for this experiment and reused if no discernible morphological and behavioural changes were detected within one week of exposure.

### Preference test

To evaluate the potential discomfort of menthol anaesthesia, a two-chamber preference test system was constructed (Abreu et al. 2016; Junior et al. 2018), allowing the fish to choose between clean or conditioned water (with the above-described anaesthetic concentrations). Briefly, a white acrylic tank (18 × 26 × 8 cm) containing sponges and fine mesh was used to maintain two separated compartments of unmixed laminar flows (Video S1 illustrates the non-mixing flows between chambers) created by two circulating pumps (0.8 L/min). This rate was adjusted so that the laminar flow did not pose a significant challenge or stress to the fish being tested. An experimental area of (18 × 10 × 8 cm) allowed the free movement of fish between compartments. The water used was the same from the maintenance tanks and 100 random fish (*n* = 10 per concentration) were individually placed into the experimental area. Following an acclimation period (150 s), anaesthetic concentrations were introduced (except for 200 mg/L due to the low recoveries observed), and videos were recorded from above by a smartphone camera (resolution: 1920 × 1080 pixels/30 frames per second). The location of the fish during the exposure period (150 s) was analysed using the ANY-maze tracking software (Stoelting Co., USA). These times were selected based on a previous study showing aversive responses of fish to anaesthetics (Readman et al. 2013). Following fish exposure, the system was manually flushed and thoroughly washed, and the anaesthetic compartment was switched, avoiding any potential laterality bias. Confirmation of unmixed laminar flux was conducted before any assay using a non-toxic coloured food dye. A negative (maintenance tank water) and a positive control using hydrochloric acid (pH 3.01 ± 0.05 (Readman et al. 2013) were also tested under the same conditions. Animals were returned to the stock tank after experimentation and reused in the following experiments.

### Short-period anaesthesia and cortisol levels

Adult fish are often anaesthetized for procedures that often require short periods of anaesthesia (Sneddon 2012; Schroeder et al. 2021). Considering this, and based on the determined anaesthetic profile of menthol, new experiments were conducted with 50 random adult zebrafish anaesthetised for a 10-minute exposure period. Initial tests were conducted with the same concentrations of MS-222 (150 mg/L) and eugenol (80 mg/L) as well as a non-aversive concentration of menthol (50 mg/L, according to the tests conducted above). However, due to the low survivability of eugenol-exposed fish after 24 h (0% survivability), its concentration was decreased to 50 mg/L. Fish (*n* = 10 per concentration) were monitored under the same conditions described for the determination of the anaesthetic profile of menthol. Individual fish were randomly placed in the acrylic tank and, after reaching stage 3 anaesthesia, zebrafish were kept in anaesthesia solution for 10 min. After this time, animals were transferred to a glass petri dish containing a sponge with a gap soaked with the same anaesthetic solution. Animals from the two controls (maintenance water and ethanol 0.2%) were sedated in 50 mg/L MS-222, as described above, to allow the collection of mucus samples without stressing the animals. While exposure to MS-222 can induce changes in cortisol levels shortly after exposure, this stress response depends upon concentration, may be species-specific (Thomas and Robertson 1991) and is time-dependent (Welker et al. 2007). In this context, while no study can be found in zebrafish, other fish studies show MS-222 to not significantly exacerbate the cortisol response until reaching stage 3 anaesthesia (Welker et al. 2007; Crosby et al. 2006). The two ends of sterile swabs were used to collect fish mucus as described by Jorge et al. (2023) and validated earlier (Jorge et al. 2024). The animals were then allowed to recover and returned to the maintenance tank. The two heads of the swab were collected in 500 µL ice-cold phosphate-buffered saline (PBS, pH 7.4) and frozen at −20 °C. For cortisol extraction, 1 mL of diethyl ether was added to the sample and allowed to stand overnight (Laberge et al. 2019) according to a previously published work (Ferreira et al. 2022). After freezing, the organic layer was transferred to a new tube and evaporated in a speed vac (Labconco Centrivap 78120-00) at 45 °C. Following evaporation, the dried samples were reconstituted in 100 µL of PBS and allowed to rest overnight. The measurement of cortisol levels was done with the DetectX Cortisol ELISA Kit (Arbor Assays, K003-H, Ann Arbor, USA), according to the manufacturer’s instructions. Cortisol levels were assayed at 405 nm with a correction at 490 nm and normalized by protein content determined at 280 nm (Noble and Bailey 2009) on a microplate spectrophotometer (PowerWave XS2, Bio-Tek Instruments, Winooski, USA).

### Statistical analysis

An a priori sample size was estimated based on the results of the cited studies, assuming a two-tailed ANOVA for independent groups to detect a medium to large effect with the power exceeding 0.8 at a significant level of 0.05. The Shapiro-Wilk and Brown-Forsythe tests were used to diagnose, respectively, the normality of distribution and homogeneity of variance in the raw data before statistical analyses. One-way analysis of variance (ANOVA) was used to compare the differences in normally distributed data, and the Tukey post hoc test was used for multiple comparisons at a significance level of 0.05. The non-parametric Kruskal–Wallis test was used if the data did not fit the normal distribution followed by the Dunn’s post-hoc test using the same significance level. The t-test was used to examine the aversive effects of anaesthetics. The data were statistically analysed using the GraphPad Prism 9.1 software (GraphPad Software Inc., San Diego, USA).

## Results

### Menthol induction and recovery

**Fig. 2:**
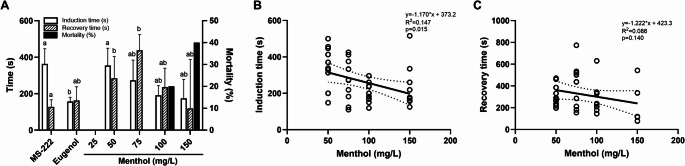
Induction and recovery times induced by different concentrations of menthol and associated mortalities (**A**). The values are presented as median and interquartile ranges from ten independent replicate exposures. Different letters indicate significant differences between groups (*p* < 0.05). Relationship between induction time (**B**) or recovery time (**C**) and varying concentrations of menthol in zebrafish. Linear regression lines and 95% confidence interval bands, with regression statistics, are presented. The 25 mg/L concentration is not shown as no deep anaesthesia was obtained

The mean times for induction of anaesthesia and recovery at different concentrations of menthol are presented in Fig. 2A. No anaesthetic effects or sedation were observed in the fish exposed to ethanol or the control fish. When exposed to 25 mg/L of menthol, fish did not reach a deep anaesthesia state, although sedative effects were observed (Supplementary Table 1). For this reason, these groups are not shown in Fig. 2A. Increasing concentrations of menthol (50, 75, 100, 150, and 200 mg/L) resulted in anaesthesia being reached faster. However, concentrations equal to or above 100 mg/L resulted in low recoveries after anaesthesia, reaching 90% mortality in the fish anaesthetized with the highest menthol concentration. Compared to MS-222, only 200 mg/L could induce a faster deep anaesthesia state (*p* = 0.010). As for eugenol, fish exposed to 50 mg/L of menthol took longer to reach a similar anaesthetic depth (*p* = 0.006) while no significant differences were observed at higher concentrations. Recovery time was longer at the 50 and 75 mg/L concentrations compared to MS-222 and eugenol, although this difference was not statistically significant. However, the recovery time decreased at higher concentrations, although no statistical differences were noted concerning MS-222 and eugenol. In addition to these results, there was a strong regression between menthol concentrations and induction time (R^2^ = 0.190, *p* = 0.002) while there was no association between menthol concentrations and recovery times (R^2^ = 0.086, *p* = 0.088) (Fig. 2B and C, respectively).

### Cardiac and respiratory effects of menthol

**Fig. 3:**
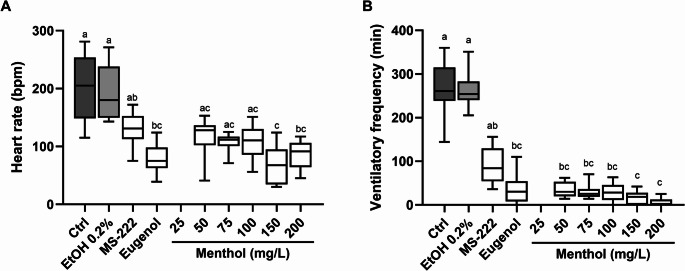
Effects of menthol anaesthesia on the heart rate (**A**) and ventilatory frequency (**B**) of zebrafish. The values are presented as median and interquartile ranges from ten independent replicate exposures and were analysed by the Kruskall-Wallis test followed by the Dunn’s post hoc test. Different letters indicate significant differences between groups (*p* < 0.05). Bpm: beats per minute

The effects of menthol anaesthesia, at the different concentrations, on the heart rate and ventilatory frequency of anesthetized adult zebrafish are shown in Fig. 3, and in Supplementary Table 1. Regarding the heart rate (Fig. 3A), ethanol exposure did not cause any interference when compared to the control group. Concerning exposure to menthol, concentrations of 75, 150 and 200 mg/L caused a significant decrease in the heart rate compared to the control group (*p* < 0.05) while no significant changes were observed for 50 and 100 mg/L, despite the decreased values. MS-222 showed values similar to the control group. The 75, 150, and 200 mg/L menthol concentrations resulted in significantly lower values, whereas the 50 and 100 mg/L concentrations also showed lower values, but these differences were not statistically significant (*p* > 0.05). No significant changes were perceived between menthol-anaesthetized fish and those anaesthetized with eugenol. Regarding the ventilatory frequency, the profile was similar to that observed in the heart rate analysis with no significant changes between menthol and eugenol, despite a decrease in the ventilatory frequency compared to the control and ethanol groups (*p* < 0.05).

### Menthol effects on behavioural parameters

**Fig. 4:**
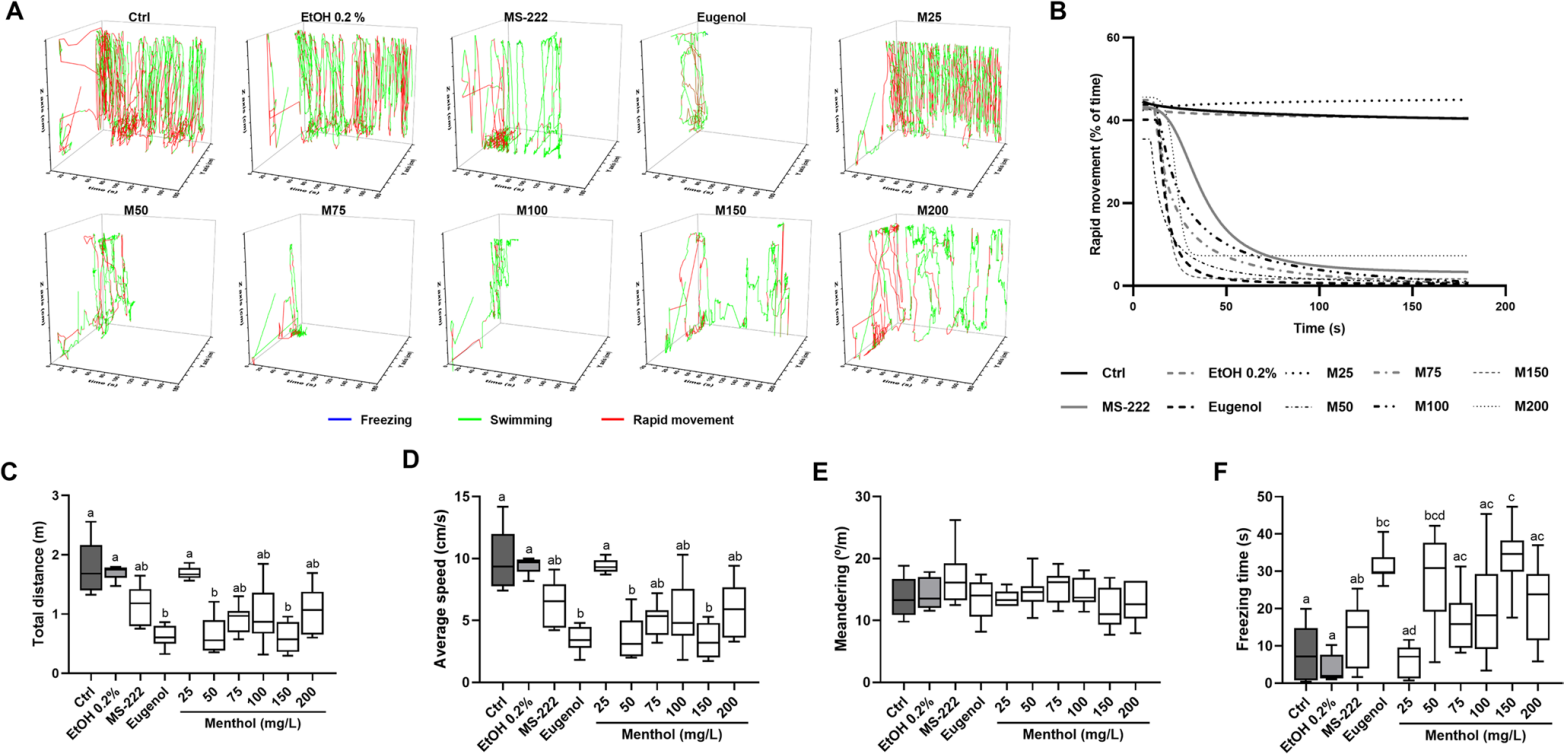
Representative three-dimensional swim trajectories of zebrafish exposed to menthol (**A**) and percentage of time the fish show rapid movement during anaesthesia induction (**B**). Locomotion profiles of zebrafish during induction: total distance travelled (**C**), average speed (**D**), meandering (**E**), and freezing time (**F**). The data are expressed as the median and interquartile ranges from ten independent anaesthesia inductions and were analysed by Kruskall-Wallis test followed by the Dunn’s post hoc test. Different letters indicate significant differences between groups (*p* < 0.05)

The behavioural features assessed during anaesthesia induction at different concentrations of menthol are presented in Fig. 4, and in Supplementary Table 2. The temporal 3D reconstructions plots from the X, Y-coordinates (Fig. 4A) revealed that menthol anaesthetized animals swam vertically and horizontally (hyperactivity) in the tank for a short period before swimming stops without rapid movements perceived. Like the control and ethanol groups, animals exposed to 25 mg/L of menthol showed signs of rapid movements in every direction of the tank. In fish exposed to the highest concentrations (150 and 200 mg/L), short bursts of swimming were noticed. In fact, as observed in Fig. 4B, animals from these groups maintained a certain percentage of swimming over time, while for the remaining menthol groups, the movement was almost null after 100 s. Regarding locomotion parameters, the data showed a significant decrease in the total distance and average speed (Fig. 4C and D, respectively) for the animals exposed to 50 and 150 mg/L in comparison to the control and ethanol groups (*p* < 0.05) while no significant changes were observed for the remaining menthol concentrations. No significant changes were observed between menthol-anaesthetized animals and MS-222 and eugenol, besides the group exposed to 25 mg/L showed no anaesthetic effect. No significant changes were observed for the meandering (Fig. 4E), while significant fluctuations were noticed for the freezing time (Fig. 4F), with menthol causing a concentration increase in the freezing time.

### Preference test

**Fig. 5:**
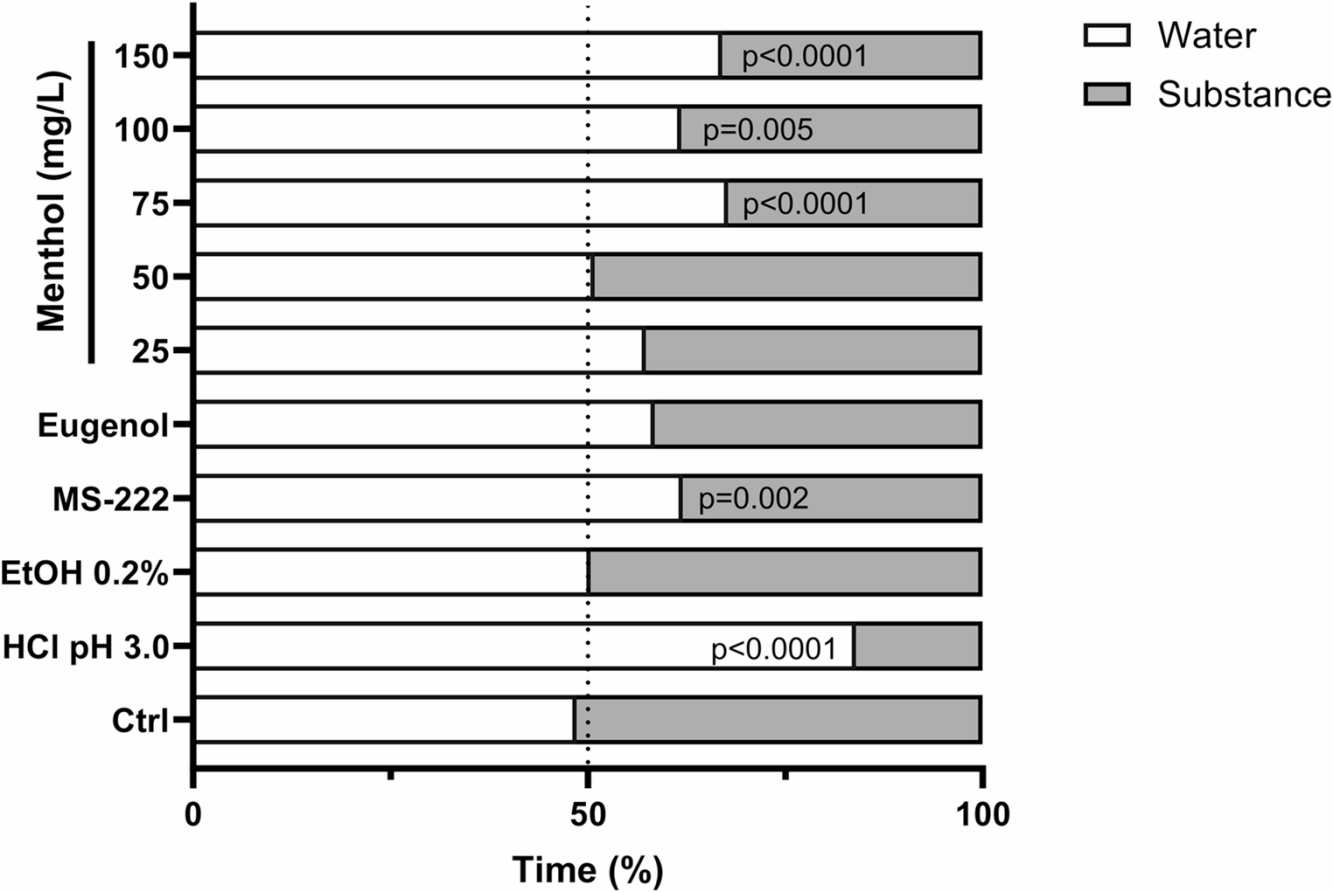
Percentage (%) of time zebrafish spent in the treated or water lane during the substance exposure period. Data are expressed as mean ± standard deviation (SD) from 10 independent experiments and p-values are shown after each bar

Figure 5 and Supplementary Table 3 show the time that animals spent in the clean or conditioned water, indicating the preference for each side. In the control and ethanol groups, no preference was detected (*p* = 0.703 and *p* = 0.912), whereas in the positive control (HCl pH 3.0), a clear aversive response of zebrafish was observed with animals standing more time in the clean zone compared to the conditioned zone (*p* < 0.0001). When menthol was introduced into the system, animals showed an aversive response to concentrations equal to or above 75 mg/L (*p* < 0.05). No significant preference was observed for the lowest concentrations of menthol as well as for the tested eugenol concentration. When MS-222 was introduced into the system, animals showed a significant preference to stay on the clean side of the system (*p* = 0.002).

### Menthol impacts on cortisol levels

**Fig. 6:**
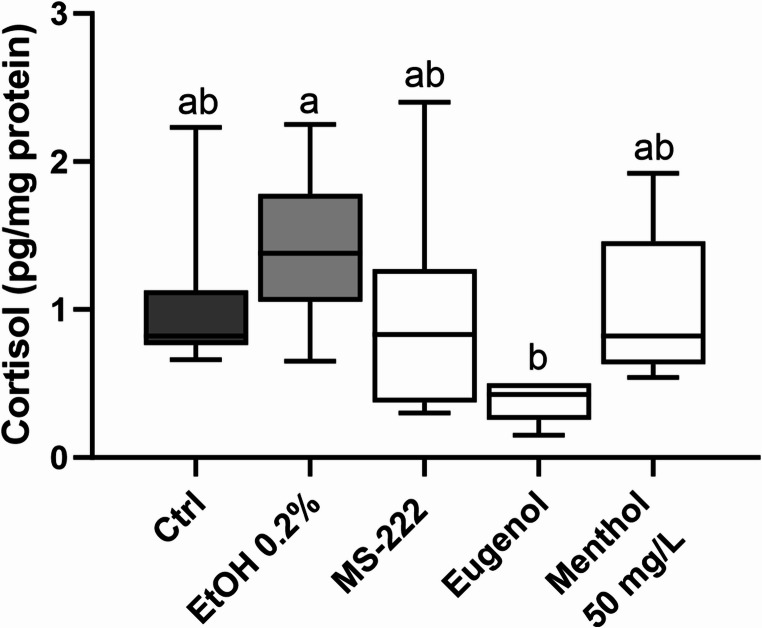
Cortisol levels in the mucus of adult zebrafish following 10-min anaesthesia. The values are presented as median and interquartile ranges from ten independent replicate exposures and were analysed by Kruskall-Wallis test followed by the Dunn’s post hoc test. Different letters indicate significant differences between groups (*p* < 0.05)

The results of the cortisol levels analysis after an extended period of anaesthesia (10 min) at 50 mg/L of menthol are shown in Fig. 6, and Supplementary Table 4. No significant changes were observed for menthol anaesthetized animals compared to control, ethanol, MS-222 or eugenol groups. Statistical differences were detected for the lower cortisol levels in the eugenol group compared to the ethanol-exposed group (*p* = 0.001).

## Discussion

There is a lack of specific anaesthetics for fish and the commonly used anaesthetics MS-222 and eugenol have been questioned (Carter et al. 2011; Barbas et al. 2021; Ayala-Soldado et al. 2023). The search for potential alternatives that respond to the welfare security requirements is therefore a necessity, with other monoterpenes being in a strategic position for this purpose (Félix et al. 2023). In this study, menthol as a new monoterpene anaesthetic for zebrafish, a model with potential application in aquaculture (Lee-Estevez et al. 2018; Ulloa et al. 2014; Jorgensen 2020; Piferrer and Ribas 2020), has been tested. The results show that menthol has a concentration-dependent anaesthetic profile like that of eugenol and MS-222. The physiological and behavioural responses were analogous among anaesthetics, although animals exposed to menthol showed less aversive response than MS-222, which was only observed at high concentrations of menthol (above 75 mg/L). When animals were exposed for an extended period of 10 min, no cortisol changes were detected following exposure to the different anaesthetics.

Owing to the species-specific responses of fish to different anaesthetics (Readman et al. 2017), concentration assessment is mandatory when testing new fish species. This allows not only to establish the appropriate depth of anaesthesia via immersion bath (usually achieved within 5 to 10 min), ensuring the safety of the tested fish species (with low mortality rates) but also allowing recovery to a normal state within an appropriate time (up to 10 min) (Neiffer and Stamper 2009). In the present study, menthol was tested for zebrafish anaesthesia, fulfilling the above-described requirements for anaesthesia, with favourable anaesthetic concentrations set ranging from 50 to 75 mg/L. A similar pattern was recently reported in adult zebrafish exposed to menthol under concentrations ranging from 60 to 120 mg/L (Baesso et al. 2024), further supporting the use of this compound to anaesthetise this fish species. In addition, similar anaesthetic profiles were reported in other adult species such as *Oligosarcus argenteus* (Uehara et al. 2019) as well as in *Oncorhynchus mykiss* (Teta and Kaiser 2019), *Lophiosilurus alexandri* (Ananias et al. 2022), *Piaractus brachypomus* (Zapata-Guerra et al. 2020) and *Aulonocara nyassae* (Ferreira et al. 2020) juveniles. However, higher concentrations (> 100 mg/L) are required to anaesthetise *Poecilia reticulata* (da Cunha et al. 2020) as well as zebrafish larvae (Vieira et al. 2024), highlighting the need for species- and stage-specific research. The menthol anaesthetic profile, at the referred range, was comparable to that observed for MS-222 and eugenol and also to that described in the literature (Collymore et al. 2014; Jorge et al. 2021; Grush et al. 2004; Baesso et al. 2024), further supporting its anaesthetic use. Interestingly, the lowest concentration (25 mg/L) of menthol did not induce the state of deep anaesthesia, although it showed potential sedative effects, as previously described for other species (Teta and Kaiser 2019; Uehara et al. 2019; Medeiros Junior et al. 2018; Sepulchro et al. 2016). Overall, this might be interesting for procedures where fish need to be sedated, such as transportation. Aside from these results, there was a correlation for induction time, although no correlation between menthol concentration and recovery time was observed. This has been reported for other adult species (Teta and Kaiser 2019; Uehara et al. 2019; Ferreira et al. 2020; Baesso et al. 2024). On the other hand, a linear correlation has been detected for induction and recovery times in juvenile stages (Pereira-da-Silva et al. 2016). This counterintuitive stage-specific outcome may be explained by the lipophilic properties of monoterpenes, which allow them not only to adhere to and rapidly penetrate adult body tissues, as well as be promptly eliminated from the tissues (Félix et al. 2023), a feature that might be desirable when anaesthetising fish species. Moreover, additional factors influencing anaesthetic response in adults, such as the individual physiological variability (e.g., metabolic rate, gill function, among others), may also contribute to the observed variability despite stable external conditions like pH, temperature, and body size. In addition, menthol anaesthesia caused cardiorespiratory depression, as observed in other studies (Ananias et al. 2022; Cantanhêde et al. 2021; Alho da Costa et al. 2022). It is worth noting that this is a common feature of fish anaesthesia (Soldatov 2021), previously described as transient and reversible for different monoterpenes (Félix et al. 2023). Yet, there is the risk of hypoxia during short-term exposures to high concentrations of menthol (> 100 mg/L), resulting in high mortalities and, therefore, high concentrations should be avoided for this species. Actually, and even though the experiment did not include an un-anaesthetised control group, changes in the hypoxia-inducible factor 1 subunit alpha mRNA levels have been described following eugenol and menthol fish anaesthesia (Zapata‐Guerra et al. 2020), requiring additional supporting experimental evidence.

Apart from these effects, menthol sedation has been associated with a potent modulation of GABA_A_ receptors (Watt et al. 2008; Kasai et al. 2014) which, in turn, promotes changes in the central nervous system function (Weir et al. 2017). In the current study, the lowest menthol concentration caused no significant behavioural changes, while the behavioural differences for the control group during the induction phase with the highest menthol concentrations were relatively narrow. It is worth noting that an initial period of hyperactivity was observed for all drugs before a reduced activity was noted. This event has been previously described when fish are subjected to anaesthetic baths of both natural and synthetic drugs (Pereira-da-Silva et al. 2016; Uehara et al. 2019; Ross and Ross 2009) and is likely due to the irritating nature of the anaesthetic agents (Readman et al. 2013). It should be noted that MS-222 behavioural effects, during anaesthesia induction, were negligible compared to the control groups, a phenomenon already described in this species (Nordgreen et al. 2014). Nevertheless, for the higher concentrations of menthol tested, short bursts of swimming were noticed, which do not favour their application for fish anaesthesia, as reported above. This may be regarded as an anxiety or panic response, with the fish trying to reduce the flow of menthol through the gills. Still, meandering was not affected by any drug or concentration, but the freezing time increased with monoterpenes, although a high variability was noted. Overall, the results support the faster anaesthetic effects of these compounds as the swimming activity of fish under anaesthesia is expected to decrease while freezing periods increase. Additionally, while no aversive responses were noted for the lowest menthol concentrations (≤ 50 mg/L), the animals exposed to concentrations exceeding this threshold showed aversive behaviour towards menthol. While no previous study could be found in the literature with a similar approach in fish, one study has found essential oils, whose major fraction is constituted by monoterpenes, to not induce aversive or attractive behaviours in fish (Junior et al. 2018). It is worth noting that ethanol and eugenol did not elicit an aversive behaviour, while MS-222 caused the fish to change their lane preference, similar to what was previously reported in zebrafish (Readman et al. 2013) and other species (Readman et al. 2017). Although the underlying mechanism requires further elucidation in zebrafish, the present results indicate the use of 50 mg/L of menthol as a potential alternative for anaesthetic purposes in adult zebrafish.

Regardless of the aversive behaviour, fish exposed to different stressors, such as anaesthetic drugs, may experience alterations in cortisol levels (Ferreira et al. 2022; Le et al. 2019a). Cortisol has long been recognised as a stress response indicator in fish (Wendelaar Bonga 1997). In the present study, no significant variations were noticed in cortisol levels quantified in the mucus of animals anaesthetised for 10 min with different anaesthetics compared to the control group. Although no study could be found in the literature with a similar approach, the correlation with plasma cortisol levels has been previously described (Carbajal et al. 2019). Hence, menthol anaesthesia has been shown to attenuate the stress responses in fish species (Pereira-da-Silva et al. 2016), while the responses of other anaesthetised species have been described as drug-dependent and observed after a period of time (peak plasma cortisol levels are detected around 30 min post-stress, decreasing thereafter) (da Cunha et al. 2010; Zahl et al. 2010; Palić et al. 2006). These differences can be attributed to the procedure used in the control group, which cannot be discarded as a factor biasing the results. In fact, most of the referenced works analyse cortisol levels in un-anaesthetised animals that are manually restrained and exposed to air for blood collection. Per se, these operations are described as stressors that activate the hypothalamic-pituitary-interrenal (HPI) axis, resulting in cortisol release (Ghisleni et al. 2012; Ramsay et al. 2009). Yet, when animals are anaesthetised before cortisol analysis, the reports are conflicting, with synthetic anaesthetics increasing cortisol levels while natural anaesthetics decreasing or maintaining their levels (Small 2003; Cunha et al. 2010; Zahl et al. 2010). Regardless of these results, and despite no un-anaesthetised control group being involved in the analysis, variations in the glucocorticoid receptor mRNA levels were reported following 50 mg/L menthol and 40 mg/L eugenol anaesthesia (Zapata-Guerra et al. 2020). This receptor regulates many physiological processes, such as the stress response (Dinarello et al. 2020). Overall, a further comprehensive investigation is required to better understand the relationship between monoterpene anaesthesia and HPI axis activation in fish species.

In conclusion, based on the findings of this study, a menthol concentration of 50 mg/L can be suggested as a useful and alternative anaesthetic for zebrafish, while higher concentrations may compromise fish welfare. In general, the anaesthetic profile was similar to that observed with MS-222 and eugenol, and no significant behavioural signs of aversion or cortisol alterations were noticed. In addition to its behavioural and physiological profile, menthol’s physicochemical characteristics and formulation strategy such as pre-dissolution in ethanol should be considered when considering its practical deployment, especially in larger aquaculture systems or long-term exposures. Like menthol, eugenol also requires pre-dilution in ethanol to ensure proper solubilization and homogeneous dispersion in water, highlighting the need for handling care with natural anaesthetics. On the other hand, while widely used, MS-222 has its own limitations, as being an acidic compound, it often requires buffering to avoid water quality disturbances. In this context, natural anaesthetics may offer a more neutral and potentially less disruptive alternative, supporting more sustainable and welfare-oriented anaesthetic practices in aquaculture and biomedical research. Notwithstanding, further studies on the physiological effects of menthol in this species (e.g. haemato-biochemical parameters, pharmacokinetics and neuronal impacts) are needed to better understand the functional effects and safety of menthol anaesthesia in fish. Currently, there is a lack of data regarding menthol’s metabolism, distribution, and clearance in zebrafish, which are critical components of pharmacokinetic understanding and essential for fully assessing its safety and efficacy as an anaesthetic.

## Supplementary Information

Below is the link to the electronic supplementary material.

Supplementary Material 1 (DOCX 25.4 KB)

Supplementary Material 2 (AVI 8.18 MB)

## Data Availability

Data is provided within the manuscript or supplementary information files.
