# Supplementary material for: Menthol as a sustainable alternative anaesthetic for adult zebrafish (Danio rerio)
Source: Vet Res Commun. 2025 Aug 26;49(5):288. doi: 10.1007/s11259-025-10860-3 (PMC12380966; doi:10.1007/s11259-025-10860-3)
Supplement: Supplementary file 1 — Supplementary Material 1 (DOCX 25.4 KB) [file 11259_2025_10860_MOESM1_ESM.docx]

|  | **Concentration (mg/L)** | **Time (seconds)** | | **Heart rate (bpm)** | **Ventilatory frequency (min)** | **Time (seconds)** | | | **Fish mortality (%)** | |
| --- | --- | --- | --- | --- | --- | --- | --- | --- | --- | --- |
|  |  | **A2** | **A3** |  |  | **R1** | **R2** |  | |  |
|  | 0 | - | - | 205 [149-254] ^a^ | 261 [239-315] ^a^ | - | - | 0 | |  |
| EtOH | 0.2 % | - | - | 180 [150-238] ^a^ | 254 [240-283] ^a^ | - | - | 0 | |  |
| MS-222 | 150 | 126 [86-161] ^ab^ | 363 [293-446] ^a^ | 131 [113-153] ^a^ | 84 [54-130] ^ab^ | 46 [33-102] ^a^ | 128 [79-167] ^a^ | 0 | |  |
| Eugenol | 80 | 18 [16-22] ^ac^ | 157 [132-180] ^b^ | 75 [63-98] ^bc^ | 31 [8-55] ^bc^ | 104 [73-158] ^ab^ | 163 [123-238] ^ab^ | 0 | |  |
| Menthol | 25 | 585 [464-665] ^b^ | > 600 | - | - | - | - | 0 | |  |
|  | 50 | 15 [9-28] ^c^ | 354 [285-449] ^a^ | 128 [102-137] ^abc^ | 30 [20-37] ^bc^ | 232 [168-334] ^b^ | 285 [231-404] ^b^ | 0 | |  |
|  | 75 | 14 [10-23] ^c^ | 273 [167-384] ^ab^ | 112 [101-117] ^bc^ | 26 [19-37] ^bc^ | 292 [165-421] ^b^ | 438 [204-524] ^b^ | 0 | |  |
|  | 100 | 19 [14-26] ^ac^ | 191 [164-244] ^ab^ | 111 [86-130] ^abc^ | 28 [11-46] ^bc^ | 158 [125-301] ^b^ | 236 [187-340] ^ab^ | 20 | |  |
|  | 150 | 17 [13-19] ^c^ | 174 [159-279] ^ab^ | 68 [35-125] ^c^ | 19 [0-28] ^c^ | 103 [63-372] ^ab^ | 119 [106-388] ^ab^ | 40 | |  |
|  | 200 | 19 [11-23] ^c^ | 192 [121-238] ^b^ | 92 [64-106] ^bc^ | 13 [0-13] ^c^ | 152 [152-152] ^ab*^ | 179 [179-179] ^ab*^ | 90 | |  |
| *Statistical test* | | X^2^(7)=46.33 | X^2^(6)=28.35 | X^2^(8)=54.57 | X^2^(8)=61.29 | X^2^(6)=27.49 | X^2^(6)=19.03 |  | |  |
| *p-value* | | <0.0001 | <0.0001 | <0.0001 | <0.0001 | 0.0001 | 0.0041 |  | |  |

**Table S1 - Latencies to induction and recovery stages, heart-rate, ventilatory frequency and mortality in adult zebrafish exposed to different concentrations of menthol.**

Data from ten independent replicates expressed as median and interquartile range. Statistical analysis was performed using the Kruskal-Wallis test followed by Dunn’s post hoc comparison test. Different superscripts denote significant differences between groups in the same column (p<0.05). * only one animal has recovered from this concentration.

**Table S2 - Behavioural zebrafish response to different concentrations of menthol until the loss of equilibrium (up to 180 seconds of analysis, stage A2).**

|  | **Concentration (mg/L)** | **Total distance (m)** | | **Average speed (cm/s)** | | **Meandering (º/m)** | | **Time freezing (s)** |
| --- | --- | --- | --- | --- | --- | --- | --- | --- |
|  | 0 | 1.68 [1.40-2.16] ^a^ | | 9.35 [7.75-12.0] ^a^ | | 13.3 [10.9-16.7] | | 7.20 [0.80-14.7] ^a^ |
| EtOH | 0.2 % | 1.75 [1.61-1.79] ^a^ | | 9.70 [8.95-9.93] ^a^ | | 13.5 [12.1-17.0] | | 2.00 [1.30-7.65] ^a^ |
| MS-222 | 150 | 1.18 [0.80-1.42] ^ab^ | | 6.55 [4.40-7.93] ^ab^ | | 16.2 [13.3-19.2] | | 15.1 [3.98-19.7] ^ab^ |
| Eugenol | 80 | 0.61 [0.50-0.80] ^b^ | | 3.40 [2.80-4.48] ^b^ | | 14.0 [10.6-16.2] | | 29.8 [29.2-33.8] ^bc^ |
| Menthol | 25 | 1.67 [1.61-1.78] ^a^ | | 9.30 [8.95-9.85] ^a^ | | 13.3 [12.3-14.7] | | 7.10 [1.25-9.50] ^ad^ |
|  | 50 | 0.56 [0.38-0.90] ^b^ | | 3.10 [2.10-5.00] ^b^ | | 14.6 [13.0-15.5] | | 30.9 [19.2-37.7] ^bcd^ |
|  | 75 | 0.97 [0.69-1.05] ^ab^ | | 5.35 [3.83-5.83] ^ab^ | | 16.2 [12.9-17.2] | | 15.9 [9.50-21.5] ^ac^ |
|  | 100 | 0.87 [0.67-1.36] ^ab^ | | 4.80 [3.75-7.55] ^ab^ | | 13.7 [13.0-16.9] | | 18.2 [9.20-29.3] ^ac^ |
|  | 150 | 0.57 [0.36-0.86] ^b^ | | 3.20 [2.00-4.80] ^b^ | | 11.0 [9.35-15.3] | | 34.6 [30.0-38.3] ^c^ |
|  | 200 | 1.07 [0.65-1.38] ^ab^ | | 5.90 [3.60-7.68] ^ab^ | | 12.6 [10.3-16.4] | | 23.9 [11.5-29.3] ^ac^ |
| *Statistical test* | | X^2^(9)=51.48 | X^2^(9)=51.31 | | X^2^(9)=11.92 | | X^2^(9)=48.28 | |
| *p-value* | | <0.0001 | <0.0001 | | 0.218 | | <0.0001 | |

Data from ten independent replicates expressed as median and interquartile range. Statistical analysis was performed using the Kruskal-Wallis test followed by Dunn’s post hoc comparison test. Different superscripts denote significant differences between groups in same column (p<0.05).

**Table S3 - Aversive responses of zebrafish to different concentrations of menthol.**

|  | **Concentration (mg/L)** | **Time in water (s)** | **Time in substance (s)** | **Statistical test** | **p-value** |
| --- | --- | --- | --- | --- | --- |
|  | 0 | 48.6 ± 16.1 | 51.4 ± 16.1 | t=0.387 | 0.703 |
| HCl | pH 3.0 | 84.0 ± 6.97 | 16.0 ± 6.97 | t=21.81 | <0.0001 |
| EtOH | 0.2 % | 50.4 ± 17.2 | 49.6 ± 17.2 | t=0.106 | 0.912 |
| MS-222 | 150 | 62.1 ± 14.6 | 37.9 ± 14.6 | t=3.709 | 0.002 |
| Eugenol | 80 | 58.5 ± 24.4 | 41.8 ± 24.4 | t=1.562 | 0.136 |
| Menthol | 25 | 53.7 ± 9.75 | 46.3 ± 9.75 | t=1.507 | 0.154 |
|  | 50 | 50.9 ± 18.6 | 49.1 ± 18.6 | t=0.219 | 0.829 |
|  | 75 | 67.8 ± 10.2 | 32.2 ± 10.2 | t=7.778 | <0.0001 |
|  | 100 | 61.9 ± 16.4 | 38.1 ± 16.4 | t=3.237 | 0.005 |
|  | 150 | 67.1 ± 12.8 | 32.9 ± 12.8 | t=5.979 | <0.0001 |

Values are expressed as mean ± standard deviation of 10 independent animals per replicate. Statistical analysis was made using the t-test in comparison to control group values (p < 0.05).

**Table S4 – Cortisol levels in adult zebrafish exposed to different anaesthetics during a 10 min period.**

|  | **Concentration (mg/L)** | **Cortisol**  **(pg/mg protein)** | |
| --- | --- | --- | --- |
|  | 0 | 0.82 [0.76-1.13] ^ab^ | |
| EtOH | 0.2 % | 1.38 [1.06-1.78] ^a^ | |
| MS-222 | 150 | 0.83 [0.37-1.28] ^ab^ | |
| Eugenol | 50 | 0.43 [0.26-0.50] ^b^ | |
| Menthol | 50 | 0.82 [0.63-1.46] ^ab^ | |
| *Statistical test* | | X^2^(4)=15.63 |  |
| *p-value* | | 0.004 |  |

Data from ten independent replicates expressed as median and interquartile range. Statistical analysis was performed using the Kruskal-Wallis test followed by Dunn’s post hoc comparison test. Different superscripts denote significant differences between groups in same column (p<0.05).
